# Dental Implant Therapeutic Trends Among Dentists in Palestine: A Cross-Sectional Questionnaire Study

**DOI:** 10.7759/cureus.24301

**Published:** 2022-04-20

**Authors:** Mohammad Assaf

**Affiliations:** 1 Periodontology, Al Quds University, Jerusalem, PSE

**Keywords:** palestine, questionnaire, surgical, prosthesis, dental implants

## Abstract

Background: Dental implants require good surgical and prosthetic skills with special training for successful treatment outcomes.

Aim: To assess the knowledge of the dental practitioners in Palestine about the therapeutic trends in implant dentistry.

Materials and methods: The present study is a cross-sectional questionnaire study among dental practitioners registered in Palestinian dental association. All practitioners who consented and completely filled the questionnaire were included in the study. All participants were asked basic demographic questions, and their attitude towards basic and advanced dental implant trends was assessed. Chi-square test was used to associate a correlation between the area of location of the clinic and responses to the questionnaire (p<0.05).

Results: Most of the dental practitioners were aware about the basic implant therapeutic trends. However, since most of the practitioners included in the present study had limited years of experience, they referred the advanced implant procedures to senior practitioners.

Conclusion: Dental practitioners in Palestine have good knowledge on the dental implant therapeutic trends. However, they do not carry out the advanced implant procedures and usually refer them to senior practitioners. There is no variation in the knowledge of the implant therapeutic trends based on varying locations of dental clinics.

## Introduction

Tooth loss in permanent dentition can be associated with dental caries, periodontal disease, or trauma. This severely affects the quality of life of an individual [1]. Hence, replacement of missing teeth is of great importance to restore the oral function and provide good aesthetics for the loss in the anterior teeth region. Dental implants are currently a promising means to ensure good rehabilitation of missing teeth [2,3].

Placement of dental implants requires good surgical as well as prosthetic skills. Special training is usually undertaken by the dental practitioner to specialize in dental implants. Due to the increase in the demand for dental implants worldwide, newer trends keep emerging with new research in dental implants [4]. 

Early research on dental implants includes introduction of micro-rough titanium implant surface which replaced the machine rough surface to improve Osseo integration. Studies have demonstrated better postoperative healing and long-term success using this method [5,6]. Dental implant width and diameter largely influences the Osseo integration, thereby influencing the success of the final outcome of the dental implant [7].

Further progress in the recent trends used in dental implants include use of advanced surgical procedures like computer-guided implant surgical stents, bone augmentation, and sinus lift procedures [8]. These make the placement of the dental implants critical in places wherein replacement of teeth with implants is questionable. A good surgical knowledge and skill is required to ensure long term success of the dental implant procedures [9]. 

In addition to the choice of dental implant type, clinical knowledge on its indication and good surgical procedure, the type of prosthetic rehabilitation also influences the success of dental implants [10]. The screw retained and cement retained prosthetic rehabilitation are the most commonly used rehabilitation methods and their choice should be made by the clinician based on the clinical scenarios since each has its own advantage and risks [11].

The dental practitioner should have an in-depth knowledge about the recent evolving trends to ensure the success of dental implants. It is the clinician's judgments of the type of technique and material used which determines the long-term successful outcome. A previous study has evaluated the oral health attitude of dental implants among dental students [12]. The knowledge of dental implant practitioners on the recently developing trends has not been assessed previously. Hence, the present study aims to assess the knowledge of dental practitioners in Palestine about the therapeutic modalities in dental implants.

## Materials and methods

The present study follows a cross-sectional study design. It is a questionnaire study conducted among Palestinian dentists interested in dental implantology in the West Bank.

Dentists participating in an implantology conference were approached and asked to fill the questionnaire, and were informed about the study being conducted. All practitioners who did not agree to participate in the study and those who did not completely fill the questionnaire were excluded from the study. Ethical approval for the methodology was obtained from the Research Ethical Committee of Al-Quds University (Ref No: 90/REC/2019).

In order to avoid language bias, the questionnaire was translated into Arabic. The validity of the questionnaire was assessed among five sample evaluators prior to implementation to the final sample size.

All dental practitioners were asked for their respective specialization. They were also asked about the type of implant treatment they provided in the clinic and their years of practice providing dental implants. General demographics of the practitioners like the location of the clinic and its classification was inquired to understand the correlation of the changing trends in implants based on location.

The first three questions assessed the dental practitioners’ choice of implant trends based on different clinical scenarios like missing anterior teeth, adjacent premolars, and molars. Clinician preference for the length and width of the implant used was also included in the questionnaire. Practitioners' preference of screw-retained crowns or cemented crowns were also noted. 

Trends based on advanced technology like computer-guided stents, immediate healing caps, and open sinus lift surgeries were also noted. Their preference of placing implants immediately after extraction was also inquired. Lastly, all dentists were asked where they learnt to place dental implants.

All the data obtained was entered into a spreadsheet. Statistical analysis was done using SPSS software version 22. Frequency was calculated for all the variables. Chi-square test was done to find any correlation between individual variables. p-value less than 0.05 was considered significant. 

## Results

A total of 143 practitioners gave consent to participate in the study. Out of them only 137 gave a completely filled questionnaire for the study and were included.

Most of the practitioners included in the study (79.56%) were general dentists and implant practitioners. Out of all the practitioners included, 72.26% were members of Palestine Association of Dental Implantology. Only 29.20% of the population had experience in implant dentistry more than 10 years. Most of the practitioners (84.67%) practiced both surgical as well as prosthetic parts of the implants. It was also noted in the present study that 84.67% of the practitioners had their clinic in the city, 47.44% of the practitioners had their clinic in middle governorates, 32.12% had their clinics in southern governorates, and 20.44% had their clinic in northern governorates (Table 1). 

**Table 1:** Questionnaire about the general characteristics of the dental practitioners

Questions	Number of observation	Frequency
Do you offer implant treatments?		
Yes, only the prosthetic part	13	9.49%
Yes, only the surgical part	8	5.84%
Yes, both parts surgical and prosthetic	116	84.67%
Specialization		
General dentist and implant practitioner	109	79.56%
Specialist: endodontist, orthodontist, pedodontist, oral medicine, other	8	5.84%
Prosthodontist	5	3.65%
Oral and maxillofacial surgeon	7	5.11%
Periodontist	8	5.84%
How many years of experience do you have in dental implant practice?		
Less than three years	40	29.20%
Three years and more but less than six	31	22.63%
Six years and more but less than 10	26	18.97%
More than 10 years	40	29.20%
Are you a member of Palestinian Association of Dental Implantology?		
Yes	38	27.74%
No	99	72.26%
What is the classification of the area where your clinics are located?		
City	116	84.67%
Camp	9	6.57%
Village	12	8.76%
In which area are your clinics located?		
Middle governorates	65	47.44%
Southern governorates	44	32.12%
Northern governorates	28	20.44%

Most of the practitioners learned implantology through a short course (35.03%); a high share (33.58%) was for local extended courses or diplomas provided by Palestinian Association for Dental Implantology (23), Al-Quds University (10), or Arab American University (3). The Chi-square test showed no statistically significant correlation between the response of the practitioner and the area of practice (p>0.05). While assessing the use of advanced surgical procedures, 58.39% of practitioners said that they do not do open sinus lift surgery and usually refer to these cases (Table 2). 

**Table 2:** Questionnaire pertaining to dental implant therapeutic trends in dental practitioners

Questions	Number of observation	Frequency
In case of missing all four upper incisors (12,11,21,22), how would you probably use implants to restore these teeth?		
Four implants with single crowns	19	13.87%
Four implants with attached crowns	13	9.49%
Two implants with a four-unit bridge	81	59.12%
Three implants with four-unit bridge	23	16.79%
I would not use implants	1	0.73%
In case of missing two adjacent premolars and first molar, with sufficient amount of bone, how would you probably restore these teeth?		
Three implants with three single crowns	18	13.14%
Two implants with a three-unit bridge	88	64.23%
Three implants with attached crowns	31	22.63%
In case of missing two adjacent premolars, how do you prefer to replace these teeth?		
Two implants with two single crowns	60	43.79%
Two implants with two attached crowns	61	44.53%
One distal implant (5) with cantilever crown for (4)	8	5.84%
One mesial implant (4) with cantilever crown for (5)	7	5.11%
I would not use implants	1	0.73%
In general, the most common implant length that you use in your clinic is?		
≥13mm	8	5.84%
≥11mm and <13mm	37	27.01%
≥9mm and <11mm	92	67.15%
In general, the most common implant width that you use in your clinic is?		
≥4.7 mm	3	2.19%
≥4 mm and <4.7	18	13.14%
≥3.4 mm and <4 mm	109	79.56%
<3.5 mm	7	5.11%
Do you prefer using screw-retained crowns over cemented crowns?		
I prefer using screw-retained crowns whenever possible	99	72.26%
I prefer using cemented crowns	27	19.71%
I never use screw-retained crowns	11	8.03%
Have you ever used computer-guided implant surgical stent?		
Never	89	64.96%
1-6 times	31	22.63%
7-12 times	9	6.57%
13-20 times	2	1.46%
More than 20 times	6	4.38%
Do you usually place an immediate healing cap on the implant after surgery?		
I usually prefer to completely cover implant by soft tissue after implant surgery	80	58.39%
Whenever possible, I place immediate healing cap	39	28.47%
I usually place healing cap immediately	15	10.95%
I commonly use the single piece implants	3	2.19%
Would you prefer to place implants immediately after the extraction of molar teeth?		
Yes, whenever possible	79	57.66%
Yes, in most cases	5	3.65%
Rarely	33	24.09%
Never	20	14.60%
Do you do open sinus lift surgery?		
I have never done it, I refer these cases	80	58.39%
I have done it a few times but I still prefer to refer these cases	19	13.87%
I routinely do it for the indicated cases	38	27.74%
How did you learn to place implants?		
Short courses in implantology	48	35.03%
I learned from a senior colleague	15	10.95%
I learned by myself during practice	4	2.92%
I hold a specialty, master, or PhD degree in a field related to implantology	24	17.52%
Extended course or diploma at either Palestine Association of Dental Implantology, Al-Quds Uni, or Arab American University	46	33.58%

The present study showed that 59.12% of practitioners used two implants with a four-unit bridge in case of missing four upper incisors, while only 9.49% used four separate implants with attached crowns. In case of missing two adjacent premolars and first molar, 64.23% of practitioners placed two implants with a three-unit bridge and only 13.14% of practitioners placed three separate implants with three single crowns. In case of missing two adjacent premolars, 44.53% of the practitioners preferred placing two separate implants with two attached crowns whereas 43.79% preferred two separate implants with two separate single crowns. Chi-square tests show no statistically significant correlation in the practitioner's responses and the area of location of the clinic (p>0.05) (Table 2). 

Most of the practitioners (67.15%) used implants with a width of ≥3.4 mm and <4 mm and length between 9 mm and 11 mm. Their preference for long implants was low. Very few of them (5.84%) preferred to use implants above 13 mm. Majority of the practitioners (79.56%) used implants with the width of ≥3.5 mm and <4 mm, and very few practitioners preferred larger width of implants. The present study showed that 72.26% of practitioners preferred using screw retained crowns. Chi-square test shows no statistically significant correlation in the practitioner's response and the area of location of the clinic (p>0.05) (Table 2).

While evaluating the recent advances, it was found that 64.96% of practitioners did not use computer-guided implant sets. Most of the senior practitioners used computer-guided implant stents frequently. However, the Chi-square test showed no significant correlation in the placement of implant stents and area of location of the clinic (p>0.05) (Table 2). 

The present study also showed that 58.39% of practitioners preferred to completely cover the implant with soft tissue after the surgery, while 28.47% preferred to place the healing caps whenever possible, and only 10.95% placed healing caps immediately. While assessing the timing of the placement of dental implants, it was seen that 57.66% of practitioners preferred to place the implants immediately after the extraction whenever possible (Table 2).

## Discussion

Dental implant provides a good option for the replacement of the missing teeth. Placement of dental implants requires good surgical skill and adequate knowledge to attain optimum clinical success [13,14]. Since the science of dental implants is constantly evolving, being up to date with the current trends is of importance. As a result, the current study was carried out to assess the practitioners' understanding of current treatment trends in dental implants in Palestine.

Most of the practitioners practiced surgical as well as prosthetic parts of the dental implants. However, there were few practitioners who practiced only the surgical part; most of these were oral surgeons. There were also practitioners who did only the prosthetic part; most of them were prosthodontists. A study by Petropoulos et al showed that oral and maxillofacial surgeons were trained well in surgery whereas prosthodontists were trained in prosthesis [15].

For implant placement in the anterior region, most of the practitioners used two implants with four-unit bridges. A study by Wheeler et al has shown that in the esthetic zone, placement of two implants with four attached crowns can provide ideal implant placement [16]. In a study by Gross et al, which describes the current concepts, placement of singular implant for premolar and two implants for missing molars and premolars can provide a good success rate [17].

The availability of the bone in the case scenario is what determines the implant length and width [18]. A width of 5-6 mm of the implant should provide a good 1 mm of bone support around it [19]. In the present study, practitioners preferred less wide implants. This can be attributed to the fact that they were aware of the good bone support required for good success of dental implants. In this study, participants preferred lengths between 11 mm and less than 13 mm, which is a commonly available length of dental implants. A study by Malmstrom et al have shown that there is no difference in the success rate of short or long implant [20].

Another study which evaluates current trends in dental implants considers implant length, diameter, and biomechanical factors in the success of dental implants [21].

Most of the practitioners in the present study did not use computer-guided stents or sinus lift surgeries. This can be due to the complex nature of these procedures and very few senior practitioners were included in the study. Another study by Al-Dajani et al shows that sinus lift surgeries for implants required additional skills from the implant practitioner [22].

A study by Biggs and Litvak shows that immediate placement of gingival caps for provisional prosthesis can provide good healing of the gingival tissue [23]. However, very few practitioners used gingival caps immediately. Placement of the implant immediately after the extraction minimizes the loss of bone, but healthy bone should be available for implant placement [24]. The practitioners in the present study have followed the same idea and placed the implant immediately whenever possible. The present study shows no correlation between the type of location of the clinic and the responses of the practitioner. Similar results were obtained in another study wherein dental practitioners had knowledge about dental implants even in rural areas [25].

The present study follows a questionnaire study design to evaluate the knowledge of dental practitioners regarding implant trends. Clinical study evaluating real-time clinical success of the recent implant trends can be beneficial in long-term success of dental implants. 

## Conclusions

The present study shows dental practitioners in Palestine to have good knowledge of the current implant trends. However, most of the advanced implant procedures like sinus lift surgeries and computer guided stents are performed by senior practitioners only. In the present study, oral surgeons preferred to do the surgical part and prosthodontics preferred to do the prosthetic part due to their expertise. This study advocates placement of two implants with four-unit bridges in the anterior esthetic zone. It also considers a width of 5-6 mm with 1 mm of surrounding bone and 11-13 mm long implants to achieve good clinical outcome. Additionally, it prefers immediate placement of implant after extraction to preserve bone structure and advocates the use of gingival caps to achieve good tissue healing. Clinical studies evaluating the success rate of implant using current trends can be useful for long-term survival of the dental implant. 
